# Biological control of the native endophytic fungus *Pochonia chlamydosporia* from the root nodule of *Dolichos lablab* on Fusarium wilt of banana TR4

**DOI:** 10.3389/fmicb.2024.1371336

**Published:** 2024-03-27

**Authors:** Yunfan Zhou, Limei Yang, Shengtao Xu, Shu Li, Li Zeng, Hui Shang, Xundong Li, Huacai Fan, Si-Jun Zheng

**Affiliations:** ^1^Yunnan Key Laboratory of Green Prevention and Control of Agricultural Transboundary Pests, The Ministry of Agriculture and Rural Affairs International Joint Research Center for Agriculture, The Ministry of Agriculture and Rural Affairs Key Laboratory for Prevention and Control of Biological Invasions, Agricultural Environment and Resource Research Institute, Yunnan Academy of Agricultural Sciences, Kunming, Yunnan, China; ^2^Resource Plant Research Institute, Yunnan University, Kunming, Yunnan, China; ^3^Bioversity International, Kunming, Yunnan, China

**Keywords:** Foc TR4, endogenous rhizobia, *Pochonia chlamydosporia*, biocontrol, *Dolichos lablab*

## Abstract

Fusarium wilt of banana caused by *Fusarium oxysporum* f. sp. *cubense*, Tropical Race 4 (TR4) is a soil-borne disease, and it is devastating. At present, the biological control using antagonistic microorganisms to mitigate TR4 is one of the best strategies as a safe and green way. Yunnan has abundant and diverse microbial resources. Using the dual-culture method, the antagonistic endophytic fungi against TR4 were isolated and screened from the root nodule of *Dolichos lablab*. The effect of the highest antagonistic activity strain on the morphology of the TR4 mycelium was observed using the scanning electron microscope. According to morphological characteristics and sequence analysis, the strain was identified. The biocontrol effect and plant growth promotion were investigated by greenhouse pot experiment. Using the confocal laser scanning microscope and the real-time fluorescence quantitative PCR, the dynamics of TR4 infestation and the TR4 content in banana plant roots and corms would also be detected. In this study, 18 native endophytic fungi were isolated from a root nodule sample of *Dolichos lablab* in the mulch for banana fields in Yuxi, Yunnan Province, China. The YNF2217 strain showed a high antagonistic activity against TR4 in plate confrontation experiments, and the inhibition rate of YNF2217 is 77.63%. After TR4 culture with YNF2217 for 7 days in plate confrontation experiments, the morphology of the TR4 mycelium appeared deformed and swollen when observed under a scanning electron microscope. According to morphological characteristics and sequence analysis, the strain YNF2217 was identified as *Pochonia chlamydosporia*. In the greenhouse pot experiment, the biocontrol effect of YNF2217 fermentation solution on TR4 was 70.97% and 96.87% on banana plant leaves and corms, respectively. Furthermore, YNF2217 significantly promoted the growth of banana plants, such as plant height, leaf length, leaf width, leaf number, pseudostem girth, and both the aboveground and underground fresh weight. Observations of TR4 infestation dynamics in banana roots and corms, along with real-time fluorescence quantitative PCR, verified that YNF2217 inoculation could significantly reduce the TR4 content. Therefore, YNF2217 as *P. chlamydosporia*, which was found first time in China and reported here, is expected to be an important new fungal resource for the green control of Fusarium wilt of banana in the future.

## 1 Introduction

The banana (*Musa* spp.) is the most important fruit in tropical and subtropical regions, and it is also one of the most traded fruits in the world (Luo et al., 2023). However, in recent years, the banana industry has been restricted by various pests and diseases, among which Fusarium wilt of banana (FWB) is the most widespread and serious disease. FWB, also known as Panama disease or banana yellow leaf disease, is a destructive fungal soil-borne disease caused by *Fusarium oxysporum* f. sp. *cubense*, with Tropical Race 4 (TR4) infestation being particularly severe (Thukkaram et al., 2020; Yadav et al., 2021). The chlamydospores of this pathogen can survive in the soil for more than several decades, making control efforts challenging (Fu et al., 2016). Given the serious threats posed by FWB, effective prevention and control methods for the development of the banana industry around the world have become crucial. At present, soil fumigation, strategic crop rotation (Li et al., 2020), selection and breeding of varieties which are disease-resistant varieties (Xu et al., 2017; Huang et al., 2023), application of chemicals (Guo et al., 2013), biological control (Wang et al., 2013), and other methods are used to restrain FWB in China and other countries. However, the chlamydospores of FWB can persist in the soil for many years, limiting the effectiveness of crop rotation control. Additionally, planting disease-resistant varieties has the disadvantages of unstable yield and poor fruit flavor (Sunisha et al., 2020); and long-term chemical application will not only make the pathogen resistant but also cause environmental pollution. In recent years, application of biological strains has become the focus of FWB control because of its advantages of economic efficiency, green environmental protection. The results of the experiment reveal that biological strains can decrease TR4 while having no effect on the banana quality or the yield (Fu et al., 2017). However, the biocontrol effects of some biological strains in field experiments are not ideal; therefore, a large number of antagonistic strains that are more efficient and have adapted to the local ecological growth conditions must still be screened. At present, the strains that have been screened and have good antagonistic effects in previous studies, such as *Pseudomonas* (Li et al., 2021), *Bacillus* (Huang et al., 2017), *Trichoderma* (Qin et al., 2017), and *Streptomyces* (Zhang et al., 2021). Fan et al. (2021) screened and obtained the biocontrol bacteria *Bacillus amyloliquefaciens* YN0904 and *B. subtilis* YN1419, which were identified by our research team in the previous study. The biocontrol effects in the pot experiment were 82.6% and 85.6%. Additionally, two biocontrol bacteria could promote banana plant growth significantly (Fan et al., 2021). We also isolated *B. velezensis* strain YN1910 which had a significant control effect (78.43–81.76%) on FWB (Fan et al., 2023).

To further explore the native biocontrol resources that have a high biocontrol effect on TR4 other than *Bacillus* species, the root nodule of *Dolichos lablab* in the mulch of banana fields in Yuxi, Yunnan, China was selected to screen endophytic antagonist fungal strains in this study. The prevention and control experiment would be carried out in the greenhouse. Using the confocal laser scanning microscope and the real-time fluorescence quantitative PCR, the dynamics of TR4 infestation and the TR4 content in banana plant roots and corms would also be detected. The objective of this study is to evaluate the biocontrol potential of isolated microbes and to provide new and high-quality native biocontrol agents for control of Fusarium wilt of banana in the field.

## 2 Materials and methods

### 2.1 Isolation and screening of the antagonistic endophytic fungus against TR4

In January 2022, root nodules of *Dolichos lablab* were collected from the mulch of banana fields with Fusarium wilt of banana symptoms in Yuanjiang County (102°15′13^′′^ E; 23°59′21^′′^ N; altitude 895 m), Yuxi City, Yunnan Province, China. The root nodules of *Dolichos lablab* were washed with sterile water, which were then dried naturally indoors. Under aseptic conditions, the roots were sterilized with 75% ethanol and were cut into small pieces with a sterilized blade. After being washed 2–3 times in sterile water, the small pieces were immersed in 75% ethanol to be soaked for 30–60 s and then into 0.1% mercury chloride for 30–60 s. Next, the small pieces were washed again 2–3 times with sterile water before grinding out the sap. The endophytic fungi were obtained on the Rose Bengal medium (Rose Bengal medium: Peptone 5 g, Glucose 10 g, KH_2_PO_4_ 1 g, MgSO_4_·7H_2_O 0.5 g, Agar 20 g, Chloramphenicol 0.1 g) using spread plate and streak plate methods. All treatments were repeated 3 times and cultured at 28°C for 72 h. Then, the colonies with different morphologies were purified for 2–3 times. Finally, when the mycelium produced spores, with the help of single-spore isolation method, the single spore was cultured on the PDA medium (PDA medium: Glucose 20 g, Agar 15 g, Potato 200 g, ddH_2_O 1000 mL, pH 7), and the strain was stored at −80°C with 50% sterile glycerol for later use.

The isolated and preserved antagonistic endophytic fungi were selected based on the primary screening and secondary screening methodologies (Fan et al., 2021). They were then cultured at 28°C for 7 days. The inhibition rate was calculated using the cross method, measuring the diameter of the TR4 colonies. The computational formula is as follows:

Inhibition rate (%) = (TR4 colony diameter of control group – TR4 colony diameter of treated group)/TR4 colony diameter of control group ×100.

### 2.2 Effect of the antagonistic endophytic fungus on the morphology of the TR4 mycelium

Using the dual-culture method, the strain TR4 (15–1) was isolated, identified, and preserved by the Banana Research Group, Agricultural Environment and Resource Research Institute, Yunnan Academy of Agricultural Sciences (Zhang et al., 2018). It was then cultured with the antagonistic endophytic fungus for 7 days at 28°C. A fresh TR4 mycelium near the antagonistic strains was selected and transferred to a temporary slide with a sterilized toothpick. The effect of the antagonistic fungus on mycelia growth of TR4 was observed using the scanning electron microscope (ZEISS Sigma 300, Germany), and the fresh TR4 mycelium cultured alone was used as the control.

### 2.3 Identification of the antagonistic endophytic fungus

#### 2.3.1 Morphological observation of the antagonistic endophytic fungus

The antagonistic endophytic fungus was inoculated on the PDA to culture for 7 days at 28°C. The morphology and color of the colony were observed and recorded. The morphology of the mycelium and spore was observed using a scanning electron microscope (ZEISS Sigma 300, Germany).

#### 2.3.2 Molecular identification of the antagonistic endophytic fungus

First, the antagonistic endophytic fungus was inoculated on the PDA at 28°C, and it was cultured for 7 days. The DNA of the antagonistic endophytic fungus was extracted using the TSINGKE plant DNA extraction kit (universal type) and part of the ITS gene sequence of the antagonistic endophytic fungus was amplified by PCR using the primers ITS1/ITS4 (5′-TCCGTAGGTGAACCTGCGG-3′/ 5′-TCCTCCGCTTATTGATATGC-3′) (Chen et al., 2021), which was synthesized by Beijing Qingke Xinye Biotechnology Co., LTD. Next, the results of PCR amplification products were compared using BLAST on the NCBI website. An ITS phylogenetic tree was constructed by adopting the adjacent method with MEGA11 software.

### 2.4 Greenhouse pot experiment

In this study, the experiment was conducted in greenhouse (temperature is 25–35 °C, humidity is 50–80%) from May to October 2022. The Brazilian cultivar (*Musa* spp. AAA) was used in the pot experiment, and the transplanting substrate was supplied by Yunnan Yuxi Leshi Technology Co., Ltd. Banana plantlets of the same plant height and size with 4–5 leaves were transplanted into a plastic pot measuring 11 cm in diameter and 12 cm in height. Through the greenhouse pot experiment, the infection process of green fluorescent protein-TR4 (GFP-TR4) (Zhang et al., 2018) was observed using the confocal laser scanning microscope (Lecia TCS-SP, Wetzlar, Germany). In addition, the real-time fluorescence quantitative PCR was used to detect the content of TR4 in banana roots and corms derived from different treatments and time points, so as to further verify the effect of the antagonistic endophytic fungus on the biocontrol of banana Fusarium wilt.

#### 2.4.1 Preparation of the antagonistic endophytic fungus and TR4 and GFP-TR4 fermentation broth

First, the antagonistic endophytic fungus and the pathogens TR4 and GFP-TR4 (Zhang et al., 2018) were activated on the PDA medium at 28°C for 7 days, and the mycelium was picked and inoculated in the PDB medium. After shaking them at 28°C and 150 r/min for 7 days, the spore suspension was filtered through 4-layer sterile gauze. The 1 × 10^6^ cfu/mL antagonistic endophytic fungus and TR4 and GFP-TR4 fermentation broth were obtained by diluting the spore suspension with sterile water.

#### 2.4.2 Experiment design and method

The pot experiment included six treatments, with each treatment repeated three times. Treatment I involved 18 banana plants in each replicate, totaling 54 banana plants (12 plants were used as control to observe the infection process of TR4 in the roots and corms at 4 time points). Treatment II involved 10 banana plants in each replicate. Treatments III and IV involved 14 banana plants in each replicate respectively (12 plants were used to detect the TR4 content in the roots and corms after TR4 invasion at 4 time points). Treatments V and VI involved 4 banana plants in each replicate.

Treatment I drenched the blank PDB (PDB medium: Glucose 20 g, Potato 200 g, ddH_2_O 1000 mL, pH 7) culture solution alone. Treatment II drenched the antagonistic endophytic fungus fermentation broth alone. Treatment III drenched TR4 spore broth and blank PDB culture solution. Treatment IV drenched the TR4 spore broth and the antagonistic endophytic fungus fermentation broth. Treatment V: drenched GFP-TR4 spore broth + blank PDB culture solution. Treatment VI drenched the GFP-TR4 spore broth and the antagonistic endophytic fungus fermentation broth (Table 1). Through treatments I and II, the plant growth promotion effect of the antagonistic endophytic fungus could be calculated. Through treatments III and IV, the biocontrol effect of antagonistic endophytic fungus could be calculated. Through treatments I, III, and IV, the TR4 content in the roots and corms after TR4 invasion was detected. Through treatments I, V, and VI, the infection process of TR4 in the roots and corms was observed.

**Table 1:** Pot experiment treatment.

**Treatment**	**Description in details**
I	Blank PDB culture solution (CK)
II	Antagonistic endophytic fungus fermentation broth
III	Blank PDB culture solution + TR4 spore broth (TR4)
IV	Antagonistic endophytic fungus fermentation broth + TR4 spore broth
V	Blank PDB culture solution + GFP-TR4 spore broth (GFP-TR4)
VI	Antagonistic endophytic fungus fermentation broth + GFP-TR4 spore broth

At 4 time points after inoculation with TR4, the DNA extracted from different banana roots and corms was subjected to real-time fluorescence quantitative PCR. At 4 time points after inoculation with GFP-TR4, the roots and corms of banana plants were selected and sliced to observe GFP-TR4 infection using the confocal laser microscope based on the protocol outlined by Zhou et al. (2023).

#### 2.4.3 Investigation of biocontrol effect

After 45 days of GFP-TR4 inoculation, the biocontrol effect of the antagonistic endophytic fungus was investigated. The McKinney index (McKinney, 1923) for both leaves and corms was recorded following the protocol outlined by Fan et al. (2021), and the biocontrol effect of each treatment was calculated. The classification for disease index was shown in Supplementary Table S1.

McKinney index (%) = ∑ (number of disease plants at all levels × relative representative value)/(total number of investigated plants × highest representative value) ×100.

Control effect (%) = (CK disease index - treatment disease index)/CK disease index ×100.

#### 2.4.4 Investigation of plant growth promotion effect

To evaluate the plant growth promotion effect, the plant height, leaf length, leaf width, leaf number, pseudostem girth, and fresh weight of both aboveground and underground parts of banana plants were measured and recorded for treatments I and II. Two time points on day 1 of inoculating the antagonistic endophytic fungus and on day 45 after inoculating with TR4 were taken for measurements and recordings.

The measurement of plant height, leaf number, and pseudostem girth was taken as outlined by Fan et al. (2021). The length and width of the first expanded leaves were also measured. The fresh weight of the aboveground portion was recorded from the base of the corm, 1 cm above the ground level, and the fresh weight of the underground portion was recorded from other parts of plant after removing the aboveground part.

#### 2.4.5 Observation of TR4 infection process in roots and corms of banana plants

To monitor the infection process of TR4 in the roots and corms of banana plants, samples of these plant parts were taken, and their tissues sectioned at 1 day, 7 days, 14 days, and 45 days after inoculating with GFP-TR4. The treatment without inoculated TR4 was used as a control. The control and treated samples of roots and corms at 4 time points were repeated three times.

The roots and corms of banana plants were washed with sterile water and sterilized with 75 % ethanol by dipping and rinsing in sterile water for 3 times. Finally, the sterilized filter paper was used to absorb surface moisture. After that, the banana roots and corms were sectioned with an ultrathin blade along the horizontal and vertical directions. Then, the sectioned tissues were placed on a glass slide, dripped with sterile water, and covered with another glass slide. The sectioned tissues were stored at 4°C in a refrigerator for observation. The excitation wavelength of GFP-TR4 fluorescence is 488:500 nm−560 nm, and the excitation wavelength of plant cell wall spontaneous fluorescence is 561:570 nm−670 nm.

#### 2.4.6 Real-time fluorescence quantitative PCR for detection of TR4 content

To detect TR4 content changes in banana roots and corms after TR4 infection, on 1 day, 7 days,14 days and 45 days after inoculating GFP-TR4, the banana roots and corms were sampled for extracting plant tissue DNA (Zhou et al., 2023). The treatment without inoculated TR4 was used as the control. The control and samples of roots and corms at 4 time points were collected and each time point was repeated three times. Using the real-time fluorescence quantitative measurement, the number of genomic quantification of gene copy was carried out. Additionally, a specific protocol and a method were referred from Zhou et al. (2023) and a standard curve was established for data analysis (efficiency, 90%−110%; correlation coefficient, R2 > 0.99).

### 2.5 Data analysis

All data were analyzed and processed using Excel 2003 and SPSS 10.0 software. One-way ANOVA was employed to calculate the difference in the mean value of each treatment. The mean value is recorded as “mean ± standard error (S.E.).” Duncan's new complex range method was employed to examine the significance of the difference among all treatments (*P* < 0.05).

## 3 Results

### 3.1 Isolation and screening of antagonistic endophytic fungus

**Figure 1:**
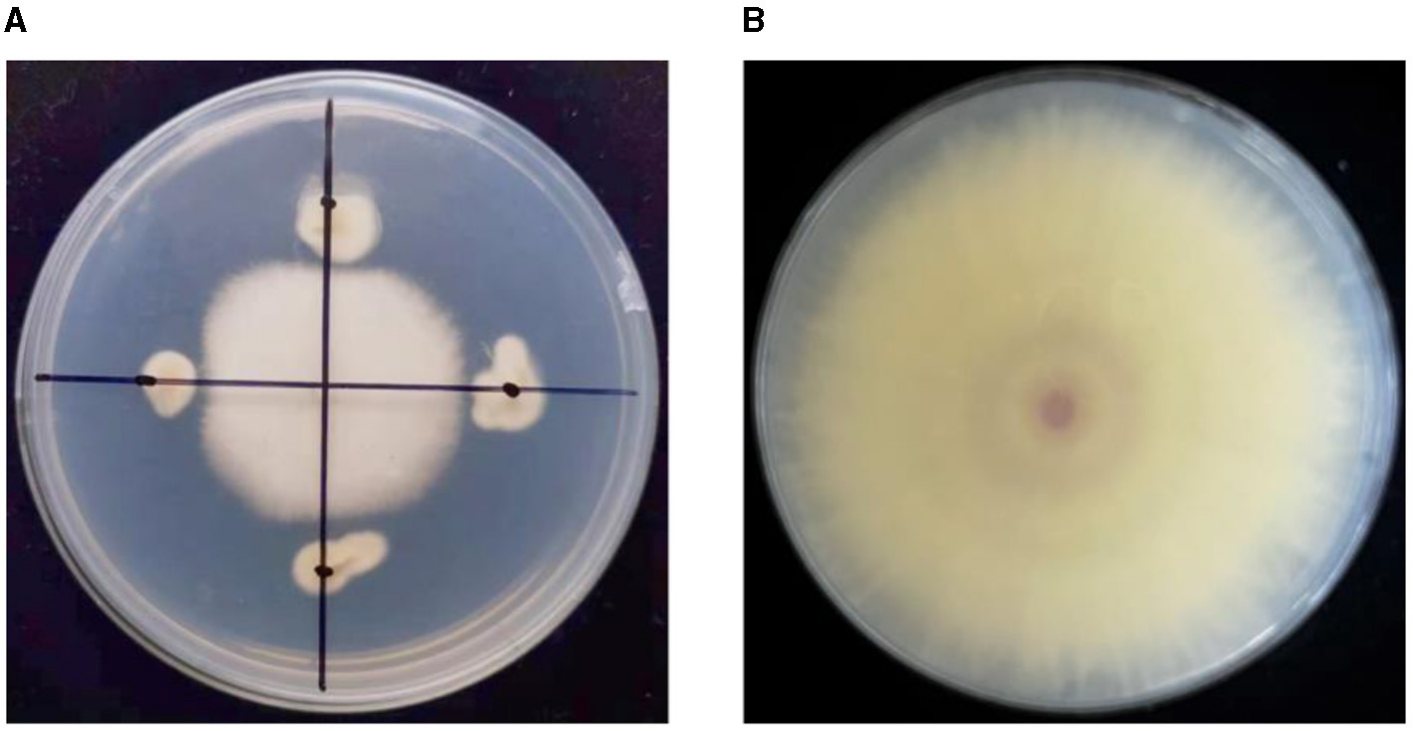
The antagonistic effect of the strain YNF2217 on TR4. **(A)** The antagonistic effect of the strain YNF2217 on TR4. **(B)** TR4 (CK).

Eighteen endophytic fungi were isolated from a root nodule sample of *Dolichos lablab* in the mulch for banana fields in Yuxi, Yunnan Province, China. After primary screening, three endophytic fungi were screened, which exhibited antagonistic activities against TR4. The secondary screening results showed that one isolate has a high antagonistic activity and is named as YNF2217. The average diameter of the TR4 mycelium was 1.98 cm, and the inhibition rate was 77.63% (Table 2; Figure 1).

**Table 2:** The antagonistic activity of the strain YNF2217 against TR4.

**Strain**	**TR4 colony diameter (cm)**	**Inhibition rate (%)**
YNF2217+TR4	1.98 ± 0.03b	77.63 ± 0.3
TR4	8.87 ± 0.03a	

### 3.2 Effect of the antagonistic strain YNF2217 on the TR4 mycelium morphology

**Figure 2:**
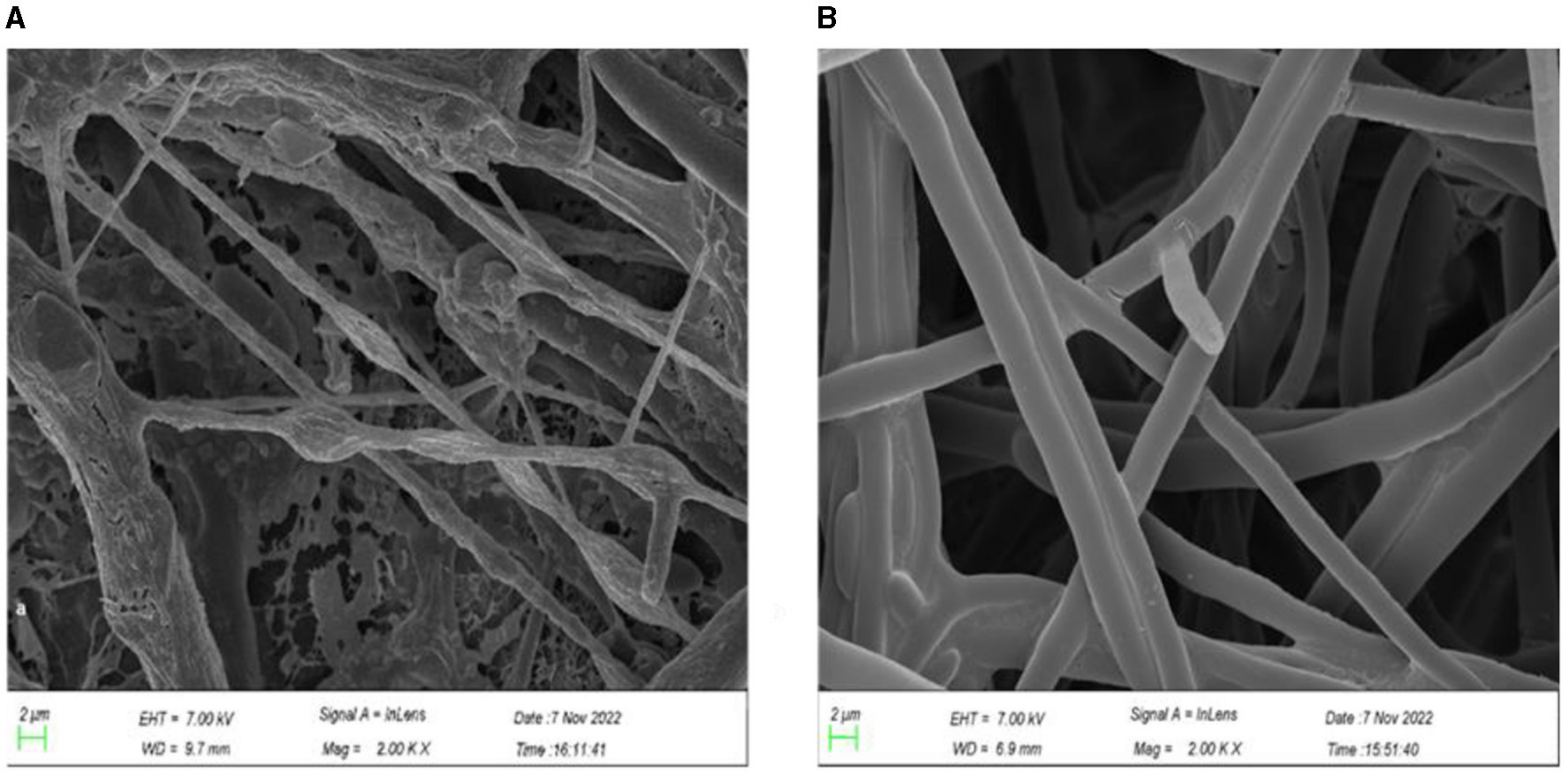
The effect of strain YNF2217 on the TR4 mycelium morphology. **(A)** The TR4 mycelium morphology under the scanning electron microscope after 7 days of YNF2217+TR4 culture. **(B)** The morphology of the TR4 mycelium under the scanning electron microscope after 7 days of TR4 culture.

After TR4 was dual-cultured with the antagonistic strain YNF2217 for 7 days at 28°C. It was discovered using the scanning electron microscope that all of the TR4 mycelium was deformed and swollen and that the interior protoplasm was concentrated and aggregated (Figure 2A). However, the mycelium of theTR4 control was of normal smoothness, uniform thickness, and natural extension, and the interior protoplasm was normal (Figure 2B).

### 3.3 Identification of the antagonistic strain YNF2217

#### 3.3.1 Morphological characteristics of the antagonistic strain YNF2217

**Figure 3:**
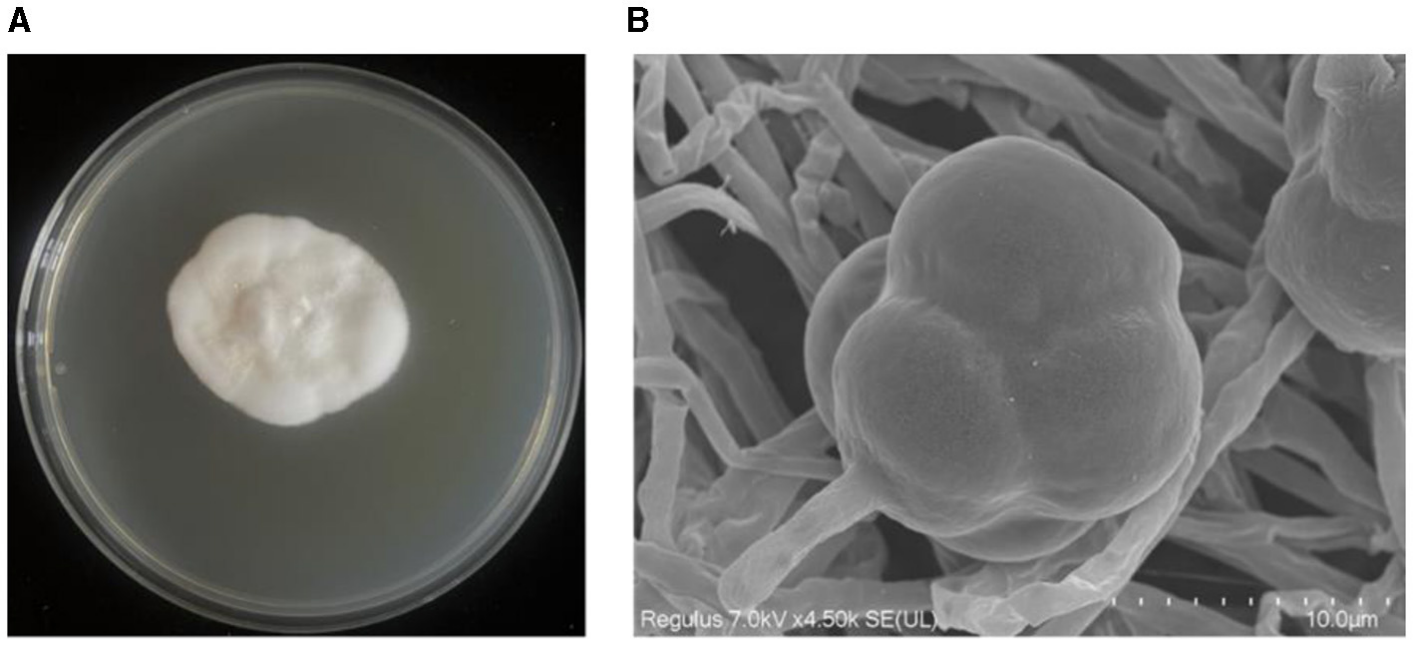
Morphological characteristics of the strain YNF2217. **(A)** The morphology of YNF2217 cultured on the PDA medium for 7 days. **(B)** The morphology of the mycelium and the conidium under the scanning electron microscope after 7 days of YNF2217 culture.

The strain YNF2217 was cultured on the PDA medium at 28°C for 7 days, the colony appeared milky white and cottony. The margin of the colony was anomalous and the colony itself was of prostrate growth. The aerial mycelium was well developed and dense (Figure 3A). Meanwhile, conidiophores were observed growing on the top or lateral branches of the mycelium through the scanning electron microscope (SEM). The conidium was individually clustered on the top of the conidiophore, and the surface of the conidium was raised and appeared like verrucae (Figure 3B).

#### 3.3.2 Molecular identification of the antagonistic strain YNF2217

**Figure 4:**
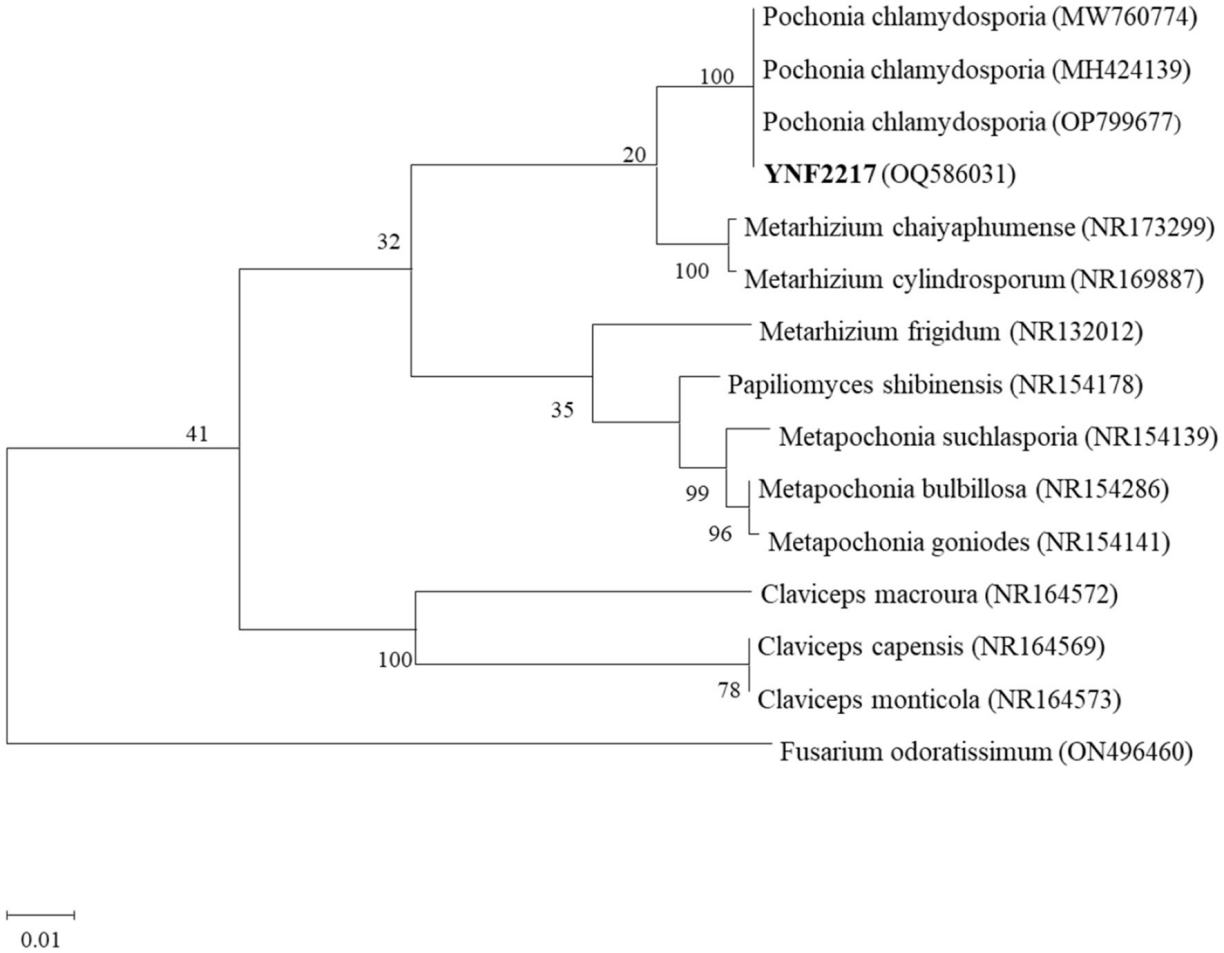
A gene phylogenetic tree of YNF2217.

Using the genome DNA of the strain YNF2217 as a template and ITS4/ITS5 as a primer, PCR amplification sequencing was performed. The ITS gene segment of YNF2217 was 1420 base pairs (bp). The ITS sequence of YNF2217 (GenBank Accession OQ586031) was compared with sequences in GenBank, and a phylogenetic tree of the sequence was constructed using MEGA 11.0 (Figure 4). It was indicated that the similarity between the sequence of YNF2217 and *Pochonia chlamydosporia* (MW760774, MH424139, OP799677) was 100%. According to the combined morphology and molecular identification methods described above, the antagonistic strain YNF2217 was identified as *Pochonia chlamydosporia*.

### 3.4 Pot experiment of the antagonistic strain YNF2217

#### 3.4.1 Biocontrol effect of the antagonistic strain YNF2217

**Figure 5:**
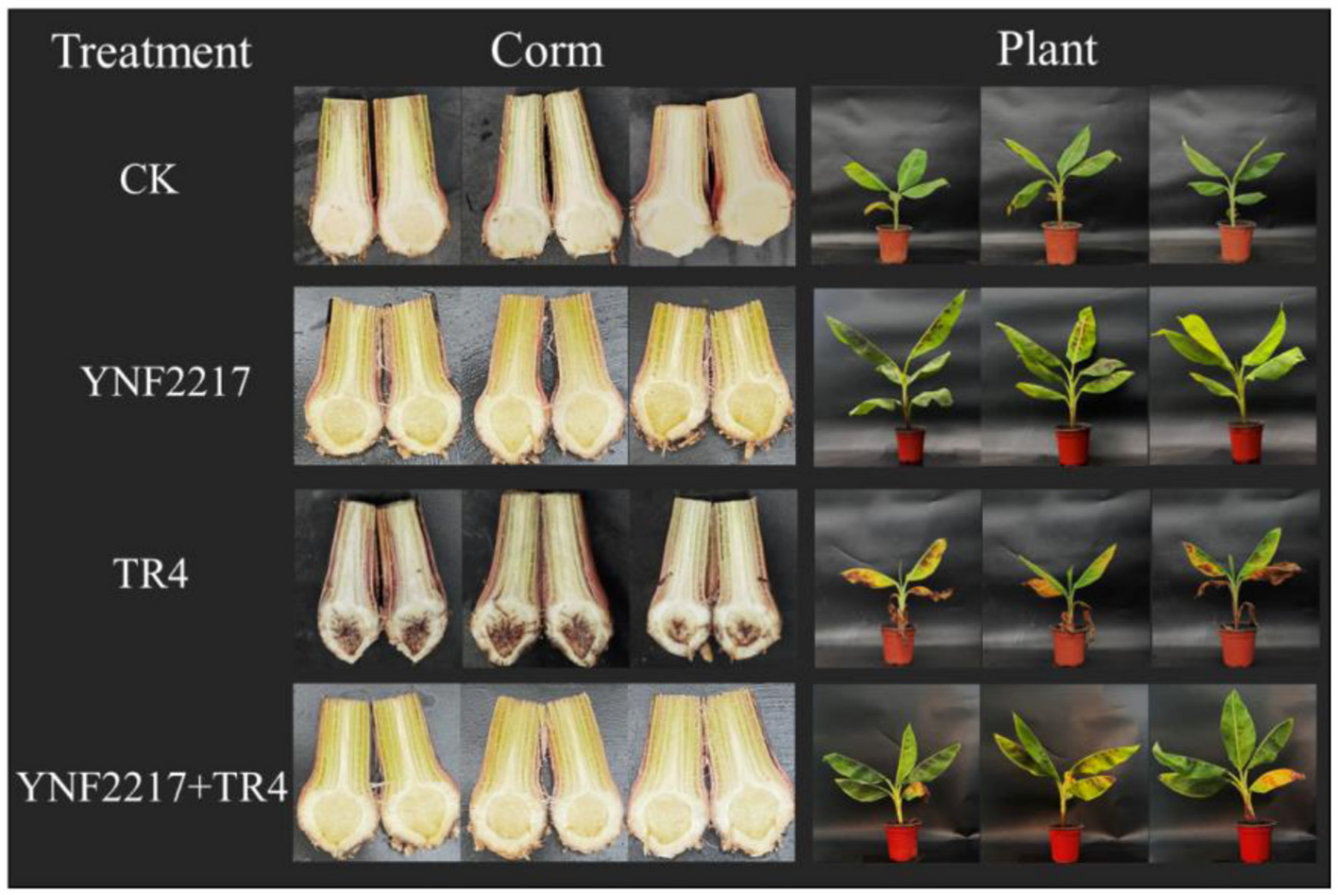
Biocontrol and plant growth promotion effects of the antagonistic strain YNF2217.

After drenching the antagonistic strain YNF2217 fermentation broth and the GFP-TR4 spore broth into banana plants for 45 days, the leaves appeared wilted and yellow, and the plants grew slowly in treatment III, which were only inoculated with TR4 (Figure 5). However, in the treatment IV of TR4+YNF2217, the most leaves remained healthy, the plants grew well and taller than those in treatment III. Additionally, after dissecting the corms, the corms inoculated with the YNF2217 were hardly invaded by the TR4 mycelium, and only a few brownish-black parts of corms were visible. However, many corms only inoculated with TR4 were brownish-black.

The disease index of the leaves and corms of banana plants in treatments III and IV was calculated 45 days after inoculation. In the treatment IV of TR4+YNF2217, the results showed that the disease index was 19.17 ± 0.83 in leaves and 2.50 ± 1.44 in corms. In the treatment III of TR4, the disease index was 66.67 ± 3.63 in leaves and 77.50 ± 4.33 in corms. In the treatment IV (YNF2217+TR4), the disease index of the leaves and the corms was significantly lower than that in treatment III (CK+TR4). According to the calculation, the biocontrol effect of the antagonistic strain YNF2217 was 70.97% in leaves and 96.87% in corms (Table 3).

**Table 3:** Control effect of the antagonistic strain YNF2217 on TR4.

**Treatment**	**Disease index**		**Control effect**	
	**Corm**	**Leaf**	**Corm**	**Leaf**
YNF2217+TR4	2.50 ± 1.44b	19.17 ± 0.83b	96.87 ± 1.86	70.97 ± 2.67
TR4	77.50 ± 4.33a	66.67 ± 3.63a		

#### 3.4.2 Plant-promoting effect of the antagonistic strain YNF2217

After 45 days of inoculated YNF2217, plant growth parameters in all 4 treatments were measured (Table 4). The banana plant height (20.97 ± 0.76 cm), pseudostem girth (20.86 ± 0.61 mm), fresh weight of the aboveground (58.64 ± 3.03 g) and fresh weight of the underground (45.85 ± 2.45 g) of the antagonistic strain treated were significantly higher than the control (CK) (17.65 ± 0.59 cm, 16.83± 0.44 mm and 33.90± 1.57 g, 27.80± 0.96 g, respectively). Moreover, the leaf length (CK: 21.45 ± 0.50 cm, YNF2217: 25.29 ± 0.75 cm), leaf width (CK: 11.00 ± 0.36 cm; YNF2217:12.45 ± 0.32 cm), and leaf number (CK: 4.73 ± 0.16; YNF2217: 6.13 ± 0.17) were significantly high. The results revealed that the antagonistic strain, YNF2217, had significant growth-promoting effects on banana plants (Table 4 and Supplementary Figures S1–S7). Furthermore, the antagonistic strain, YNF2217, also significantly promoted plant growth even in the presence of TR4 inoculation.

**Table 4:** Growth promotion effect of the antagonistic strain YNF2217 on banana plants.

**Treatment**	**Plant height**	**Leaf length**	**Leaf width**	**Leaf number**	**Pseudostem girth**	**Fresh weight of the aboveground**	**Fresh weight of the underground**
CK	17.65 ± 0.59c	21.45 ± 0.50b	11.00 ± 0.36b	4.73 ± 0.16b	16.83 ± 0.44b	33.90 ± 1.57b	27.80 ± 0.96b
YNF2217	20.97 ± 0.76a	25.29 ± 0.75a	12.45 ± 0.32a	6.13 ± 0.17a	20.86 ± 0.61a	58.64 ± 3.03a	45.85 ± 2.45a
TR4	15.30 ± 1.90d	20.68 ± 0.38b	10.24 ± 0.35b	3.30 ± 0.17c	15.25 ± 0.31c	32.77 ± 1.44b	21.50 ± 0.68c
YNF2217+TR4	19.20 ± 1.31b	24.47 ± 0.40a	10.80 ± 0.30b	4.60 ± 0.10b	17.91 ± 0.31b	53.03 ± 1.96a	41.97 ± 1.14a

#### 3.4.3 Monitoring TR4 infection in roots and corms

**Figure 6:**
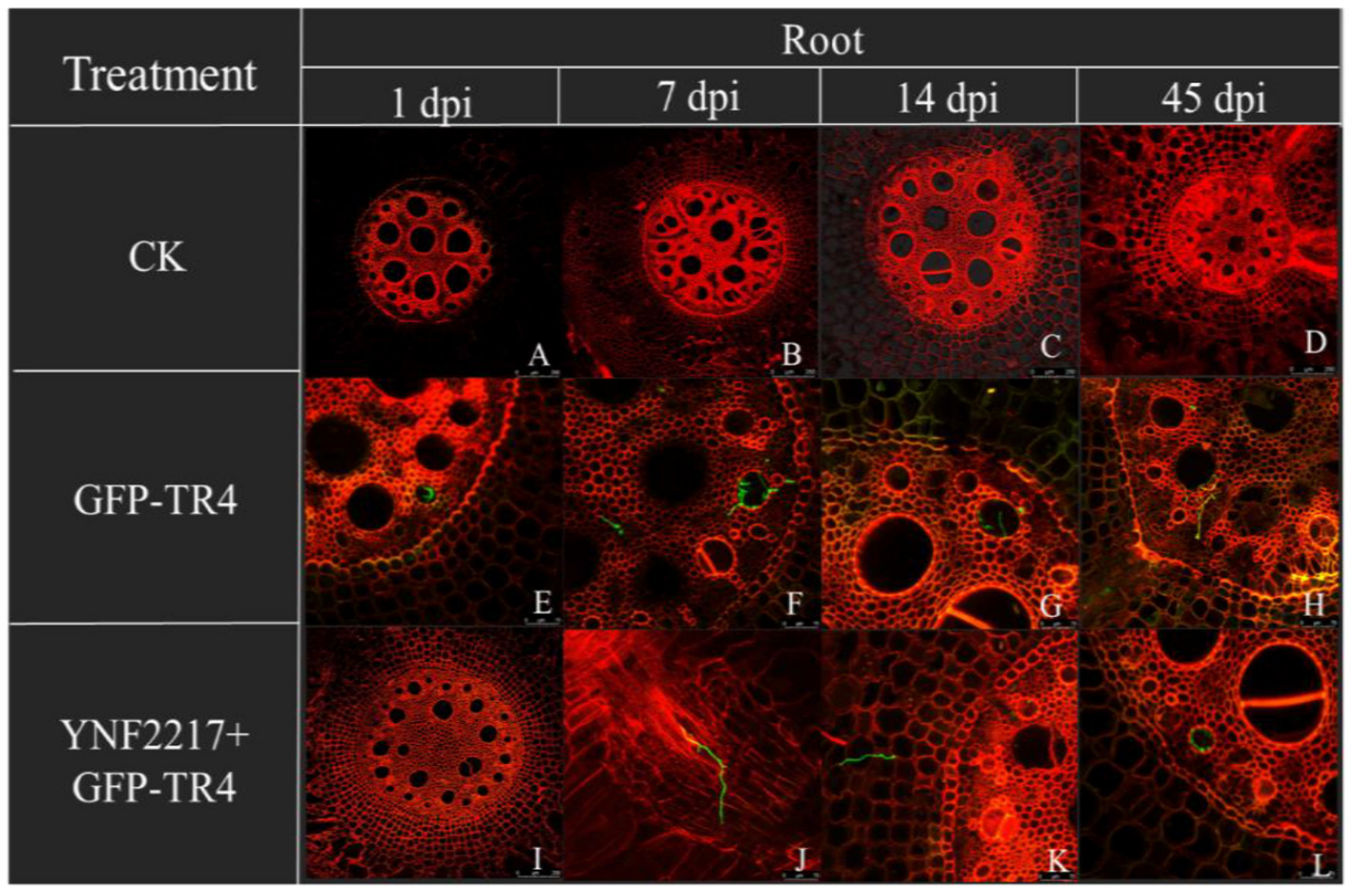
Infection process of GFP-TR4 in the roots of banana (1 dpi, 7 dpi, 14 dpi, 45 dpi). Bar = 250 mm in A, B, C, D, and I; Bar = 75 mm in E, F, G, and H.

**Figure 7:**
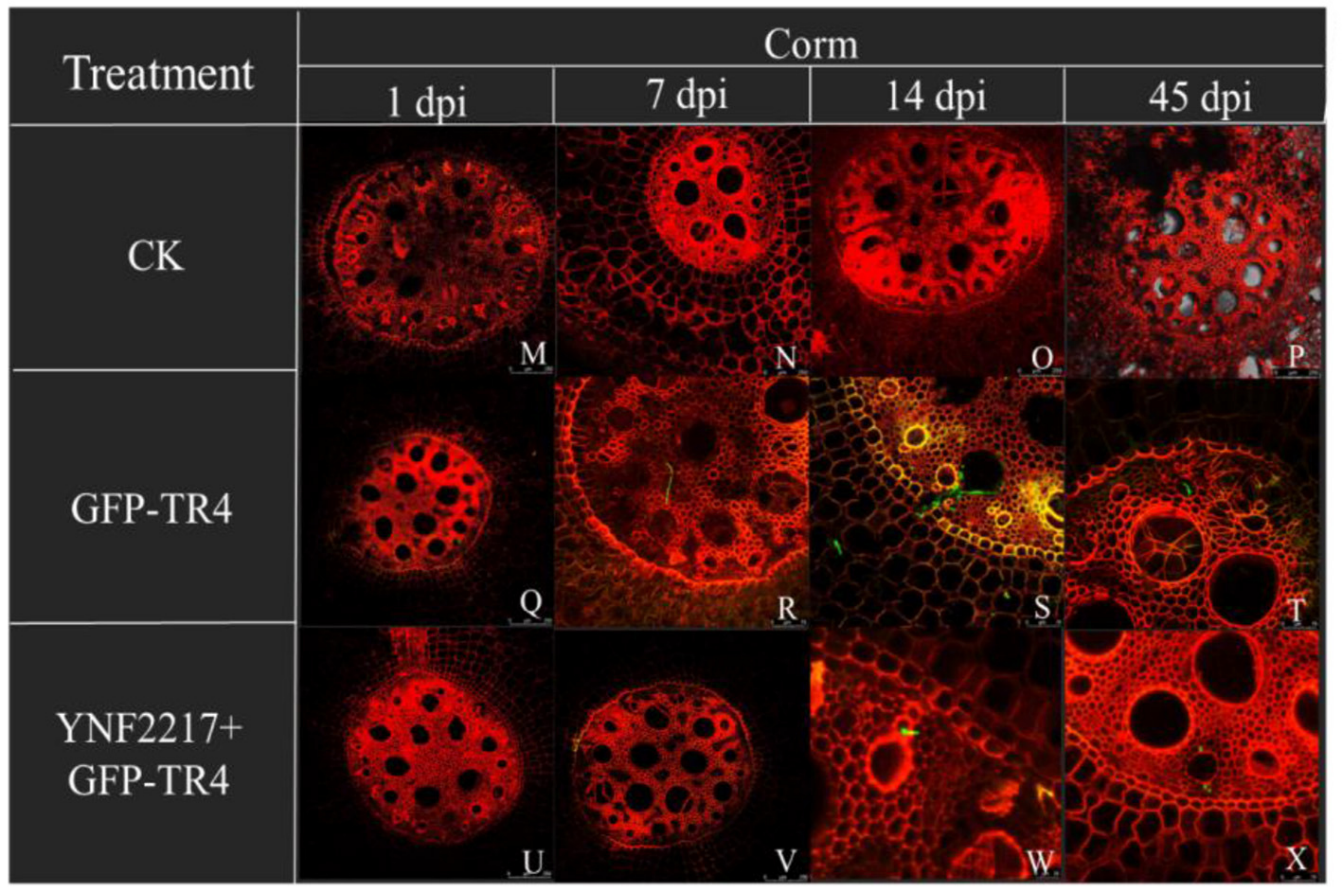
Infection process of GFP-TR4 in the corms of a banana (1 dpi, 7 dpi, 14 dpi, 45 dpi). Bar = 250 mm in M, N, O, P, Q, R, U, and V; Bar = 75 mm in R, S, T, W, and X.

The GFP-TR4 strain was used to monitor its infection process in treatments V and VI. After 1 day and 7 days of inoculating GFP-TR4, in the treatment VI (YNF2217+GFP-TR4), there were no detection of traces of the GFP-TR4 mycelium in the roots and corms. However, in the treatment V of only inoculating GFP-TR4, the mycelium was found in both roots and corms. Furthermore, after 14 days and 45 days of inoculating GFP-TR4, a small amount of the TR4 mycelium were found in the roots and corms treated with the antagonistic strain, YNF2217, while a large amount of the TR4 mycelium were present in both the roots and corms inoculating with GFP-TR4 (Figures 6, 7).

#### 3.4.4 TR4 measurement using real-time fluorescence quantitative PCR

**Figure 8:**
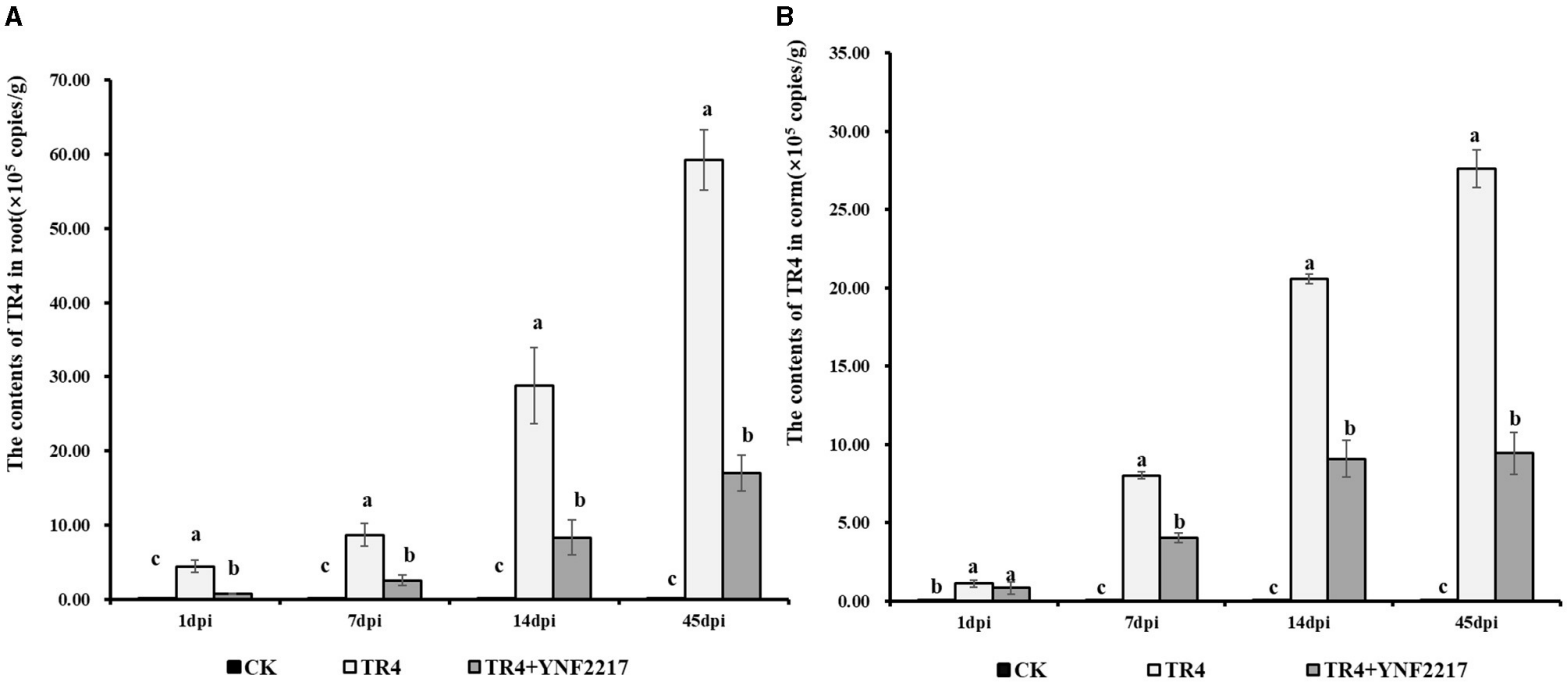
The content of TR4 in banana plants at 4 time points. **(A)** The content of TR4 in banana roots at 4 time points. **(B)** The content of TR4 in banana corms at 4 time points. The data were expressed as mean ± standard error, *P* < 0.05 was considered indicative of a significant difference, and different letters indicated significant differences.

Real-time fluorescence quantitative PCR was employed to quantify the TR4 content in roots and corms on day 1, day 7, day 14, and day 45 after TR4 inoculation. The result is shown in Figure 8, in 4 different time points, and the content of TR4 in roots after TR4 inoculation ranged from 4.41 × 10^5^ copies/g to 54.28 × 10^5^ copies/g. While, in the YNF2217 +TR4 treatment, it ranged from 0.70 × 10^5^ copies/g to 17.02 × 10^5^ copies/g. For all 4 time points, the content of TR4 in corms after TR4 inoculation ranged from 1.11 × 10^5^ copies/g to 27.61 × 10^5^ copies/g. However, in the YNF2217 +TR4 treatment, it ranged from 0.83 × 10^5^ copies/g to 9.43 × 10^5^ copies/g.

In roots, the content of TR4 treated with YNF2217+ TR4 was significantly lower than that of inoculation only with TR4 in all 4 time points. In corms, on day 7, day 14, and day 45, the content of TR4 treated with YNF2217+ TR4 was significantly lower than that of corms inoculated only with TR4. These results indicate that the TR4 content both in roots and corms was significantly reduced when treated with YNF2217+TR4, compared to inoculation with TR4 alone.

## 4 Discussion

Native strains have the ability to amplify the positive effects of biocontrol strains (Chong et al., 2023). Compared to exogenous strains, native strains have the advantages of the survival rate, reproduction rate, and stress resistance (Gan et al., 2024). Although various biocontrol strains have been developed and utilized at present, biocontrol strains are easily influenced by the environment and other soil microbial communities (Wang et al., 2023). Therefore, it is imperative to screen for local, unique, and adaptable biocontrol strains that can thrive in local ecological environments and are resistant to FWB. In this study, an antagonistic endophytic fungus, YNF2217, was isolated from the mulch in banana fields in Yuxi, Yunnan, China. Antagonistic activity screening and greenhouse pot experiments confirmed that the strain YNF2217 had high biocontrol effect on banana Fusarium wilt. The inhibition rate of YNF2217 was 77.63%. According to the morphology and molecular identification, the antagonistic strain YNF2217 was *Pochonia chlamydosporia*. In the greenhouse pot experiment, the biocontrol effect of the strain YNF2217 on the leaves and corms of banana plants was 70.97% and 96.87%, respectively. It is proved that YNF2217 had a significant biocontrol effect on the banana Fusarium wilt. In addition, various characteristics of banana plants was measured, including plant height, leaf length, leaf width, leaf number, pseudostem girth, and fresh weight both in the aboveground and belowground parts. The results showed that the strain YNF2217 had significant growth-promoting effects on banana plants. Furthermore, the results from monitoring the infection process of GFP-TR4 in roots and corms indicated that the GFP-TR4 mycelium in plants inoculated with YNF2217+TR4 was lower than that in the treatment of only inoculated with TR4 on day 1, day 7, day 14, and day 45 after TR4 inoculation. Additionally, the real-time fluorescence quantitative PCR detection results also indicated that the content of TR4 in the roots inoculated with YNF2217+TR4 was significantly lower than that in the treatment of only inoculated with TR4. On day 7, day 14, and day 45, the content of TR4 in corms inoculated with YNF2217+TR4 was also significantly lower than that in the treatment of only inoculated with TR4. Therefore, it was confirmed that this fungal strain YNF2217 had a significant biocontrol effect on FWB.

It was reported that *P. chlamydosporia* exists in the microbial population of the banana planting region (Ciancio et al., 2022). The expression of related resistance genes such as *PIN II, PR1*, and lipoxygenase (*LOX*) in the host leaf was also reported after *P. chlamydosporia* was used to treat banana plants (Tolba et al., 2021). However, there is no study linking *P. chlamydosporia* to FWB. In this study, the biocontrol effect of *P. chlamydosporia* YNF2217 on FWB was first reported in China. Therefore, this strain will be an important biocontrol strain resource for the green control of FWB. YNF2217 could be used as an additional and new biocontrol strain resource along with the previously discovered strains such as *Pseudomonas* (Xie et al., 2023), *Bacillus* (Fan et al., 2021, 2023; Duan et al., 2023), *Trichoderma* (Thukkaram et al., 2020) and *Streptomyces* (Lu et al., 2022). The prevention and control mechanisms of biocontrol fungi mainly include competition (Alwathnani et al., 2012), mycoparasitism (Amira et al., 2017), antibiosis (Vinale et al., 2014), induced resistance (Chemical et al., 2012), growth promotion (López-Bucio et al., 2015), and synergistic antagonism (Schulz et al., 2013). The active resistance of plants includes resistant genes involved and induced resistance, and induced systemic resistance is usually systematic, broad-spectrum, and non-specific (Stéphane et al., 2005). The manifestation of induced resistance includes the expression and accumulation of PR protein and antibacterial enzymes in plants. PR protein is an important component of disease and pest resistance in plants and often has antifungal and insecticidal activities (Muthukrishnan et al., 2001). Jiang had discovered that the related resistance marker genes such as *PR-1, PR-2*, and *PR-5* increased expression levels after *Nigrospora* sp. inoculation into the rhizosphere of wheat (Jiang, 2016). Tijerino et al. (2011), by analyzing the genomes of three *Trichoderma* species and using high-throughput gene knockout techniques, reported that a large number of genes related to secondary metabolism were discovered, and the genes *Tbtri5* and *Tri5* for *Trichoderma* synthase were obtained. Therefore, YNF2217 is expected to have great application prospects in the biological control of FWB.

*P. chlamydosporia* is a type of nematophagous fungus, with the ability to parasitize root-node nematodes and cyst nematode eggs. It is now widely used in the control of both animal and plant nematodes (Zhu et al., 2017). *P. chlamydosporia* serves as an effective ingredient in commercial fungicides and biological agents of plant parasitic nematodes. Moreover, its liquid culture is simple and cost-effective (Uthoff et al., 2024). Ghahremani et al. had reported that *P. chlamydosporia* is the common antagonistic fungus of vegetable root-node nematodes (Ghahremani et al., 2022). Zavala et al. (2017) discovered that *P. chlamydosporia* not only controls nematode diseases but also promotes the growth of plants, it was found that *P. chlamydosporia* promoted plant growth and improved the yield through the jasmonate signaling pathway. *P. chlamydosporia* also has a good preventive effect on animal parasitic diseases. Tábata et al. (2023) found that *P. chlamydosporia* can be parasitic in nematode eggs so as to reduce the degree of gastrointestinal nematodes in horses. Stoupa et al. (2020) found that the combined application of *Arthrobotrys cladodes* with *P. chlamydosporia* could effectively reduce the infection degree of cattle gastrointestinal nematode larvae. The greenhouse experiment results from Fu et al. (2014) showed that, when nematodes and TR4 infected bananas simultaneously, the disease index of FWB was higher than that when nematodes were not present. Yin et al. (2021) conducted experiments in banana plantations, and the results also indicate that the incidence rate of FWB was positively correlated with the disease index of root-node nematodes. So the results indicated that the control of root-knot nematodes could reduce the incidence rate of FWB. Therefore, YNF2217 as the nematophagous fungus is expected to have a better biocontrol effect on FWB when applied in the field in the future. *P. chlamydosporia* could directly inhibit TR4 and root-knot nematodes (Silva et al., 2017), and that most probably reduces the wounds of banana plants by its nematophagous nature. Furthermore, the transmission route of *Fusarium oxysporum* f. sp. *cubense* is reduced, thereby decreasing the occurrence of FWB.

The control effect of a biocontrol strain in soil-borne diseases mainly depends on its successful colonization in the soil or rhizosphere, competing with pathogens for nutrients and ecological sites, while also secreting antibacterial substances into the soil (Liu et al., 2023). As a result, under the premise of effective colonization of biocontrol strains, those that have better control effects are preferred. Lin et al. (2018) performed the transcriptome analysis of *P. chlamydosporia* and revealed its parasitism capacity on egg parasitoids, which is the main mechanism for controlling plant nematode diseases. Larriba et al. (2015) performed an analysis of barley roots after *P. chlamydosporia* colonization by transcriptome changes and found that growth-promoting genes were upregulated and plant immune-responsible genes were induced. In this study, YNF2217, identified as the native endophytic fungus *P. chlamydosporia* from the root nodule of *Dolichos lablab*, demonstrated its very promising potential application in FWB management. Based on previous studies on the relevant mechanisms of *P. chlamydosporia*, we expect that YNF2217 would have similar features as other *P. chlamydosporia*. Furthermore, the exact biocontrol mechanism and its field control effect of YNF2217 will be conducted in next research. Additionally, efforts will be made to develop the biocontrol formulation with YNF2217 for application in the management of FWB in the future.

## Data availability statement

The datasets presented in this study can be found in online repositories. The names of the repository/repositories and accession number(s) can be found in the article/Supplementary material.

## Author contributions

YZ: Conceptualization, Data curation, Formal analysis, Investigation, Methodology, Software, Validation, Visualization, Writing—original draft, Writing—review & editing. LY: Data curation, Investigation, Methodology, Software, Validation, Visualization, Writing—review & editing. SX: Data curation, Investigation, Methodology, Resources, Validation, Visualization, Writing—review & editing. SL: Data curation, Investigation, Methodology, Software, Validation, Visualization, Writing—review & editing. LZ: Data curation, Investigation, Resources, Validation, Writing—review & editing. HS: Data curation, Investigation, Methodology, Validation, Writing—review & editing. XL: Formal analysis, Investigation, Methodology, Resources, Validation, Writing—review & editing. HF: Conceptualization, Funding acquisition, Investigation, Project administration, Resources, Supervision, Visualization, Writing—review & editing. S-JZ: Conceptualization, Funding acquisition, Methodology, Project administration, Resources, Supervision, Validation, Visualization, Writing—review & editing.
