# Supplementary figures and images for: Biological control of the native endophytic fungus Pochonia chlamydosporia from the root nodule of Dolichos lablab on Fusarium wilt of banana TR4
Source: Front Microbiol. 2024 Mar 27;15:1371336. doi: 10.3389/fmicb.2024.1371336 (PMC11004353; doi:10.3389/fmicb.2024.1371336)

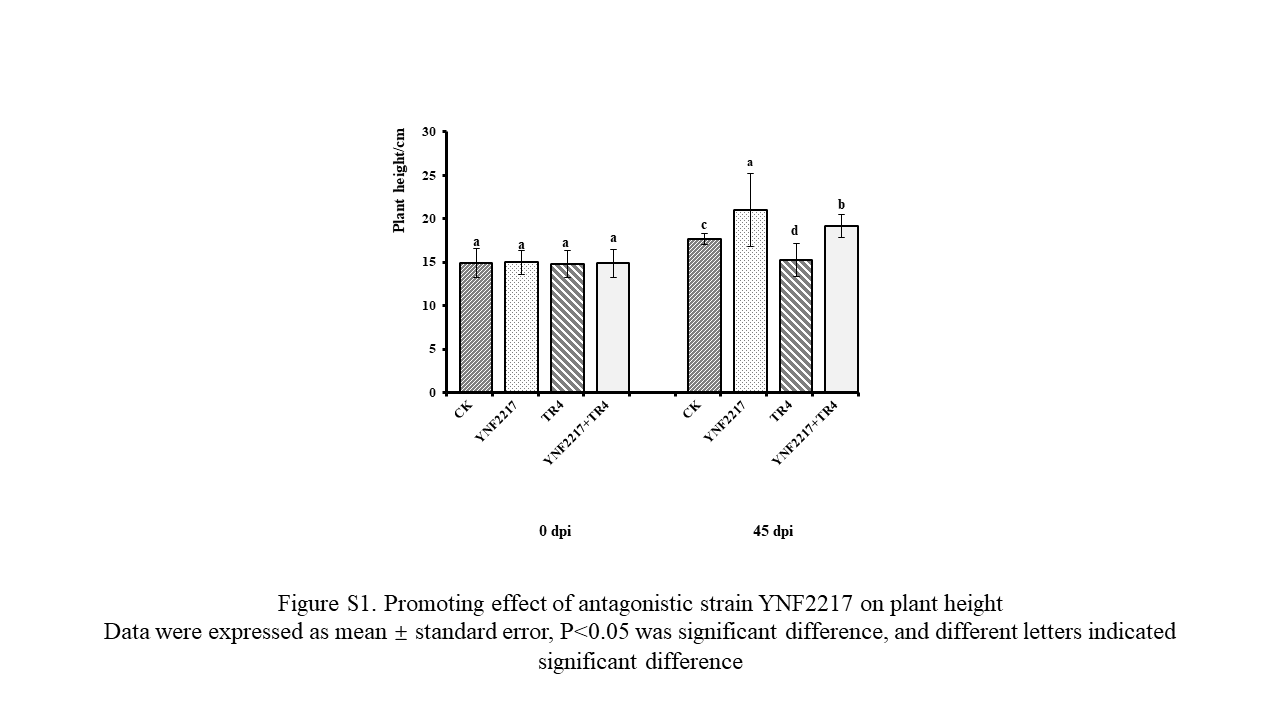

Supplement: Supplementary file 1 [file Presentation_1.zip › Figure S1.TIF]

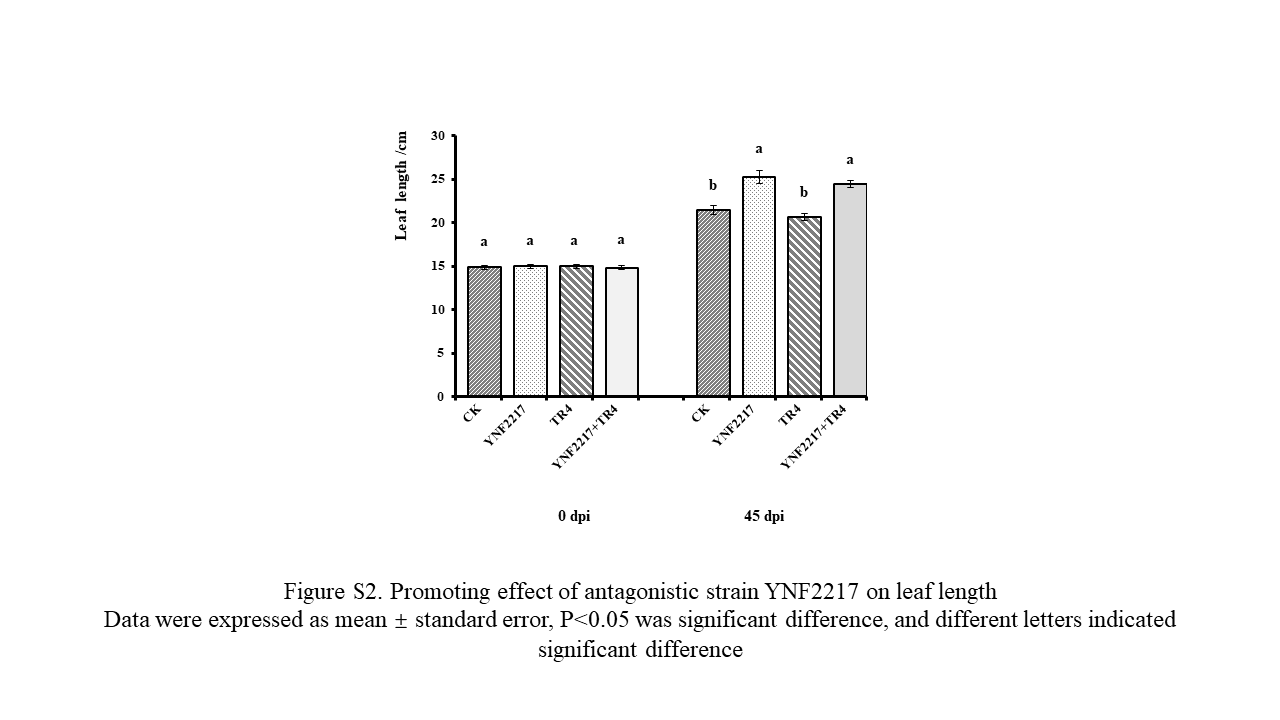

Supplement: Supplementary file 1 [file Presentation_1.zip › Figure S2.TIF]

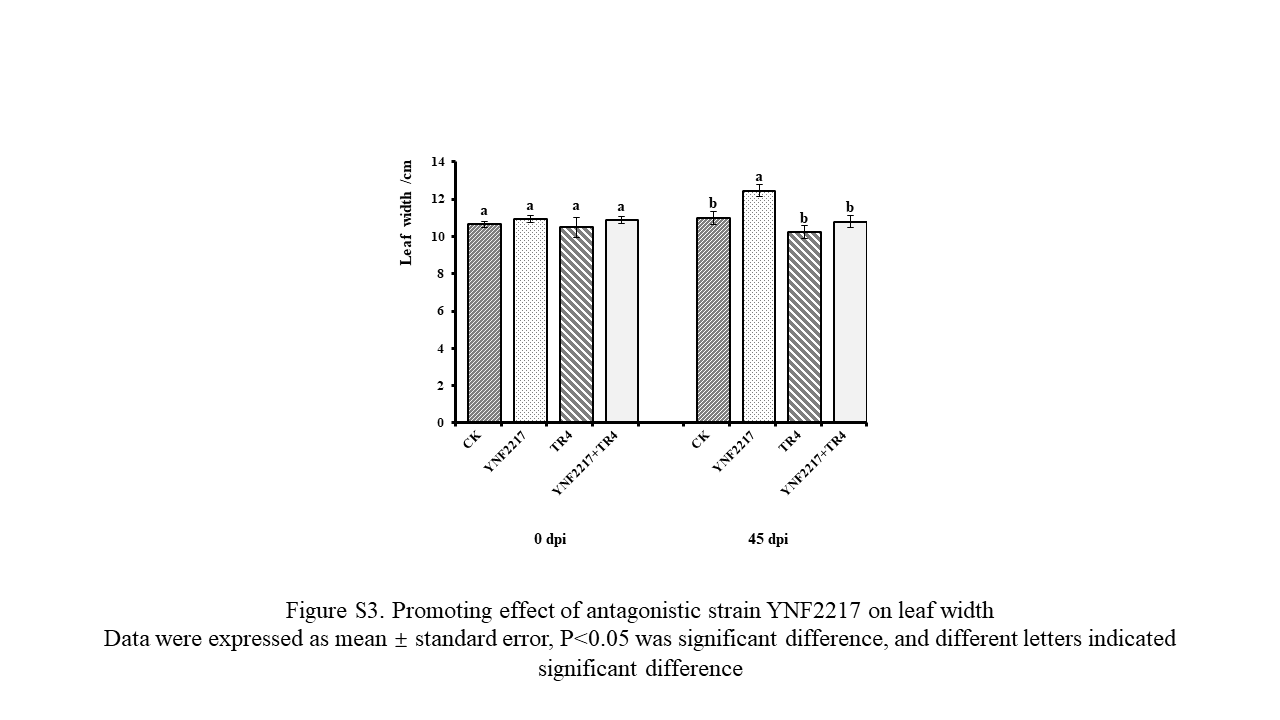

Supplement: Supplementary file 1 [file Presentation_1.zip › Figure S3.TIF]

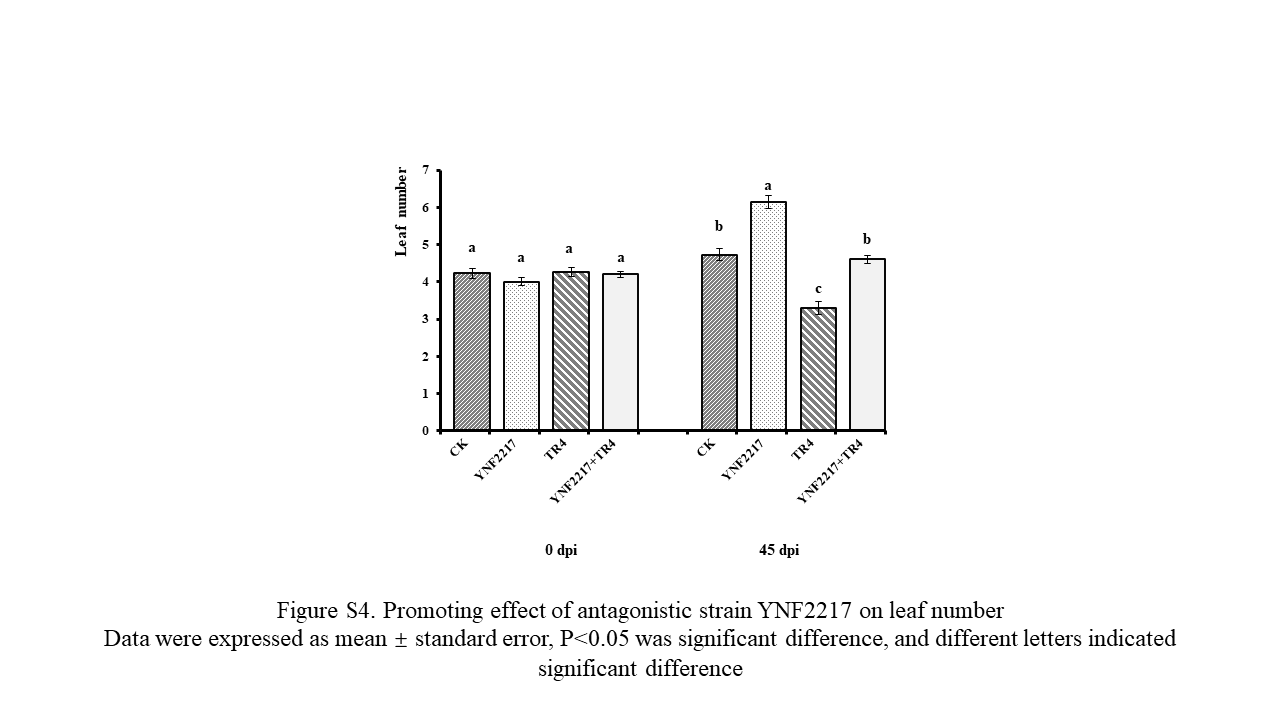

Supplement: Supplementary file 1 [file Presentation_1.zip › Figure S4.TIF]

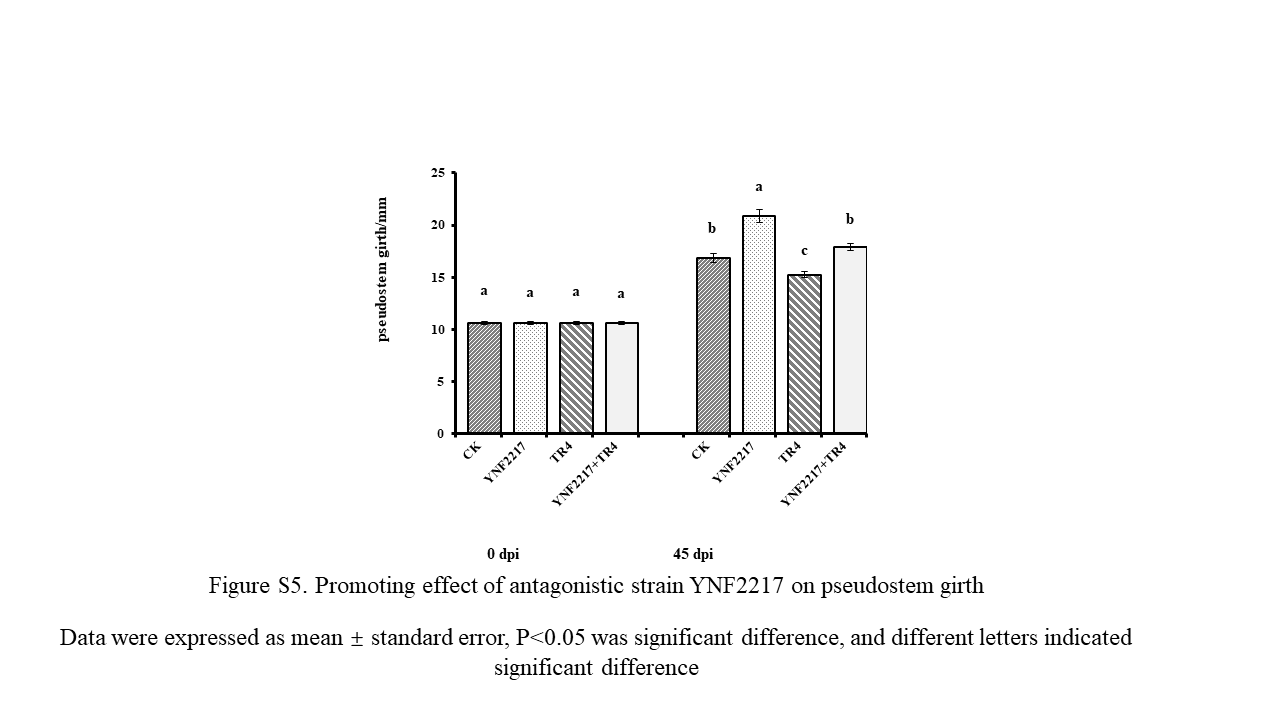

Supplement: Supplementary file 1 [file Presentation_1.zip › Figure S5.TIF]

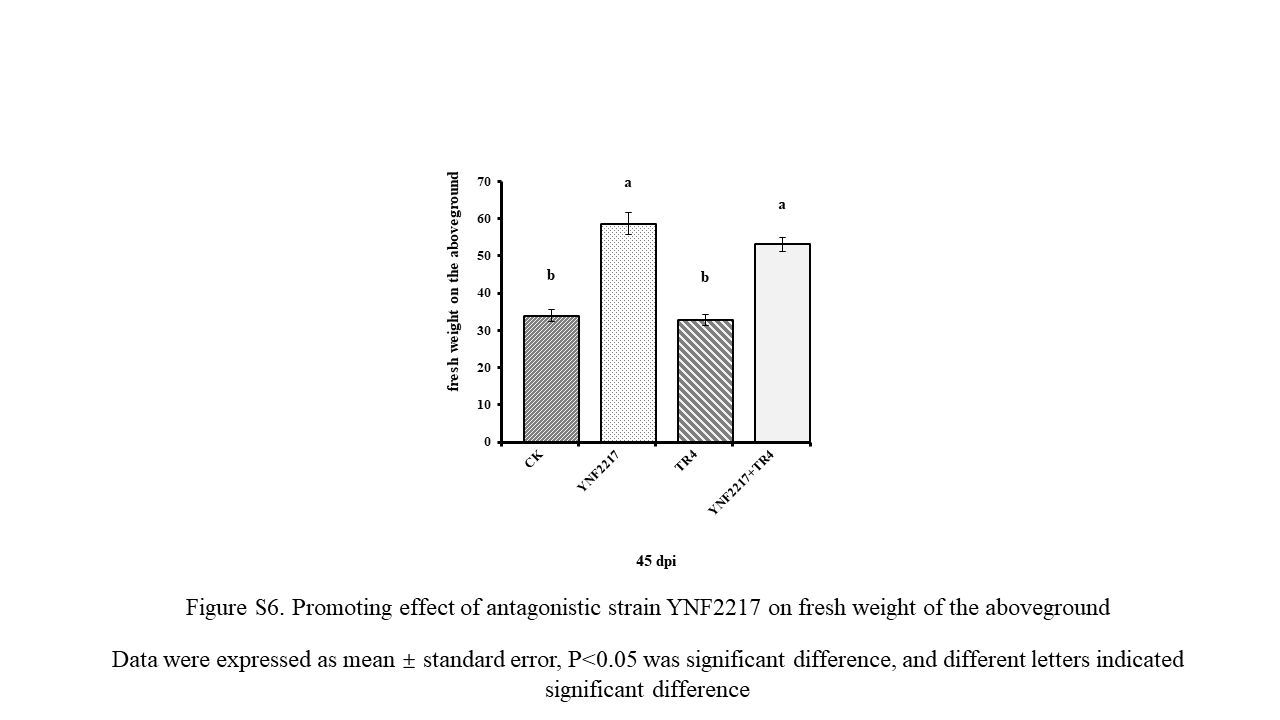

Supplement: Supplementary file 1 [file Presentation_1.zip › Figure S6.TIF]

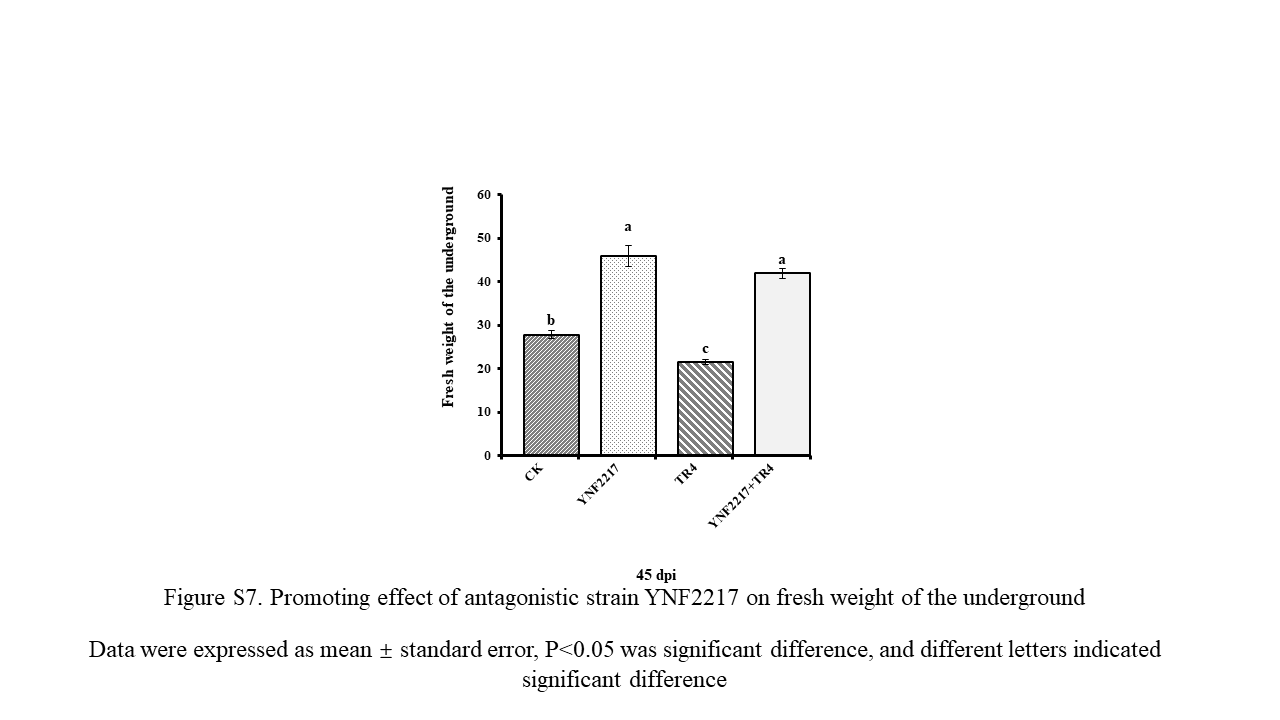

Supplement: Supplementary file 1 [file Presentation_1.zip › Figure S7.TIF]
